# Proof of Concept for Light Conducting Membrane Substrate for UV-Activated Photocatalysis as an Alternative to Chemical Cleaning

**DOI:** 10.3390/membranes8040122

**Published:** 2018-12-02

**Authors:** Lavern T. Nyamutswa, Bo Zhu, Dimuth Navaratna, Stephen Collins, Mikel C. Duke

**Affiliations:** 1 Institute for Sustainable Industries and Liveable Cities, Victoria University, Melbourne 14428, Australia; bo.zhu@vu.edu.au (B.Z.); dimuth.navaratna@vu.edu.au (D.N.); mikel.duke@vu.edu.au (M.C.D.); 2 College of Engineering and Science, Victoria University, Melbourne 14428, Australia; stephen.collins@vu.edu.au

**Keywords:** Titanium dioxide, photocatalytic membrane, water treatment, membrane fouling

## Abstract

Adopting an effective strategy to control fouling is a necessary requirement for all membrane processes used in the water/wastewater treatment industry to operate sustainably. The use of ultraviolet (UV) activated photocatalysis has been shown to be effective in mitigating ceramic membrane fouling by natural organic matter. The widely used configuration in which light is directed through the polluted water to the membrane’s active layer suffers from inefficiencies brought about by light absorption by the pollutants and light shielding by the cake layer. To address these limitations, directing light through the substrate, instead of through polluted water, was studied. A UV conducting membrane was prepared by dip coating TiO_2_ onto a sintered glass substrate. The substrate could successfully conduct UV from a lamp source, unlike a typical alumina substrate. The prepared membrane was applied in the filtration of a humic acid solution as a model compound to study natural organic matter membrane fouling. Directing UV through the substrate showed only a 1 percentage point decline in the effectiveness of the cleaning method over two cleaning events from 72% to 71%, while directing UV over the photocatalytic layer had a 9 percentage point decline from 84% to 75%. Adapting the UV-through-substrate configuration could be more useful in maintaining membrane functionality during humic acid filtration than the current method being used.

## 1. Introduction

Water scarcity affects about two-thirds of the world’s population for at least a month of every year [1]. Existing water resources of suitable quality are already over-subscribed or rapidly approaching their limits in most parts of the world. To address this gap, conveniently available poorer quality waters may be used, but they first have to be treated to meet quality standards. However, current treatments are beset with several issues, among them high costs, non-effectiveness in removing recalcitrant pollutants such as azo dyes and nitroaromatic compounds [2], and the generation of toxic secondary by-products. Improving accessibility and operational simplicity of treatment technologies would support initiatives to improve wider access to clean water.

Treatment of municipal and industrial wastewater is also becoming an important source of water for industrial and agricultural use [3]. To meet increasingly stringent water quality regulations, natural organic matter, soluble microbial products and micro-pollutants should also be removed from wastewater. Processes that have been used in tertiary water treatment processes include advanced oxidation, activated carbon adsorption, ion exchange and membrane filtration. Membrane filtration, in particular, is gaining increased use because of its lower energy footprint, compact design, lower chemical consumption and the ease with which it can be maintained and automated [3].

Membrane processes, however, have several issues which limit their use on a wider scale. One such issue is fouling, which reduces membrane separation efficiency, membrane lifespan and can raise energy costs. Maintaining performance and improving the simplicity of operation of membrane systems is necessary to make the technology more widely available to communities worldwide.

The approaches that have been used to reduce membrane fouling include pre-treating the feed, modification of membrane properties, optimisation of operating conditions, as well as optimization of module arrangement and configuration [4]. Although these reduce fouling to some extent, membrane cleaning is always employed in practice. Cleaning can be achieved hydraulically, mechanically, electrically or chemically, with the former being the most common, followed by the latter. More than one of these cleaning methods can also be applied in combination. However, these result in significant downtime and costs associated with loss of productivity, labour, energy as well as procurement, transportation, storage, and disposal of chemicals [4].

In-situ self-cleaning methods are therefore necessary to solve the fouling problem without stopping the water filtration process. One such method is coupling membrane filtration with photocatalysis. The photocatalytic properties of semiconductor photocatalysts such as TiO_2_ lead to the photo-induced partial or total decomposition of pollutants present on the surface of the membrane while photo-induced ultra-hydrophilicity leads to elimination of the remaining hydrophobic contaminants through a simple water rinsing operation. Photocatalysis and induced hydrophilicity can occur on the same surface simultaneously to give a “self-cleaning” membrane which leads to savings on cleaning procedures [5,6].

Several configurations have been used to combine filtration with photocatalysis to give an integrated hybrid water treatment process, known as a photocatalytic membrane reactor (PMR). The configurations include a slurry photocatalytic reactor with a membrane submerged in it, a slurry reactor that precedes a membrane filtration unit and a porous photocatalytic membrane in which the photocatalyst is coated onto the membrane substrate [7]. Of these three configurations, the latter is the most interesting for future water treatment because it combines both filtration and photocatalysis in one unit.

A novel configuration in which the feed solution was fed from the uncoated side of the membrane was studied, mainly to independently control the separation and photocatalytic functions of the membrane [6]. Separating the separation and photocatalysis functionalities increases process robustness because failure of one does not necessarily result in the failure of the other [8]. Another advantage cited for this configuration is retention of particulates capable of shielding UV light on the feed side of the membrane, thus making photocatalysis more efficient because the permeated side has more optical transparency than the feed side. This was envisaged to make UV disinfection of highly turbid waters more efficient in terms of UV dosage, though it could come at the expense of increased membrane fouling.

**Figure 1:**
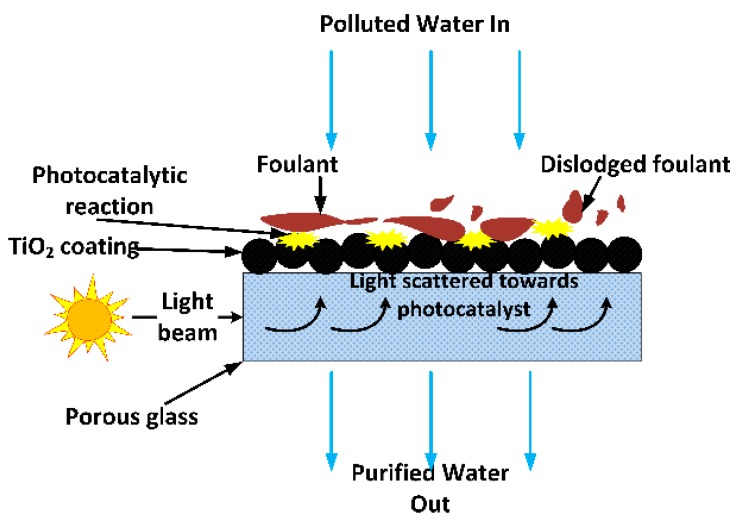
The concept of a light-conducting membrane substrate for practical implementation of a photocatalytic reaction for improved membrane performance.

To date, researchers have focused on making photocatalytic membranes for water treatment by coating nano-sized photocatalysts on opaque materials such as ceramics, organic membranes and metals. These membranes lack light transparency, requiring light to be directed through the water being treated to reach the photocatalyst coating on the substrate surface. Directing light this way in complex membrane element designs, such as ceramic monoliths, results in light attenuation before it reaches the photocatalyst coated inside the channels. Organic pollutants present in water can also strongly absorb light which was meant to reach the photocatalyst coating, reducing photocatalytic efficiency [9]. In this study, the potential of replacing these materials with optically transparent sintered glass was investigated. UV light (including solar) can then be conveniently directed through the light-transmitting sintered glass substrate to reach the photocatalyst coated on its surface. The use of sintered glass in this way was not found in the literature. If loss of light through absorption or reflection by the membrane substrate or organic pollutants present in the water being treated are minimised, it can translate to more efficient use of energy and reduced costs, and long term mitigation of fouling with minimal chemical and physical membrane cleaning. The concept is depicted in Figure 1.

The key aim of this research study was to verify the concept by coating porous glass membranes with a photocatalyst and observing ex-situ if the photocatalytic reaction can be engaged by directing light through the substrate in comparison to applying the light directly to the photocatalytic layer. The model synthetic dye, screened methyl orange (sMO), was used to observe a practical photocatalytic reaction utilising the well-known commercial catalyst, Aeroxide P25 TiO_2_. The model membrane foulant and pollutant, humic acid (HA) was used as a filtration feed solution to study the effect of applying this concept in mitigating membrane fouling.

## 2. Materials and Methods 

### 2.1. Materials

Humic acid was purchased from Fluka AG Cheische Fabrik., Buchs, Switzerland and used as a representative natural organic matter compound. Titanium dioxide P25 with 99.8% purity and composed of 80% anatase and 20% rutile phases was acquired from Evonik. The TiO_2_ had an average particle size of 30 nm and a specific surface area of 50 m^2^/g). Bovine Serum Albumin (BSA) of molecular weight 66 kDA was purchased from Sigma Aldrich (St. Louis, MO, USA). Screened methyl orange used as a model dye was purchased from Ajax Chemicals, Australia. Methanol was acquired from Chem-Supply, Australia. Nitric acid was purchased from Merck Pty Limited, Kilsyth, Australia. Acetone (99.9%) was purchased from Sigma-Aldrich, Australia. Sintered glass used as the membrane substrate was acquired from Ningbo Ja-Hely Technology Co., Ltd., Ningbo, China. This was in the form of flat circular discs of 25 mm diameter, 2 mm thickness and G5 porosity grade.

### 2.2. Apparatus

A panel consisting of 6 × 18 W UVA lamps (A.U.V.S (Ops), Pty. Ltd., Australia) with an emission peak at 365 nm was used to illuminate the membrane outside the module. The UV intensity at 365 nm was measured by a UV irradiance meter from Photoelectric Instrument Factory of Beijing Normal University, Beijing, China. A TPI 665L digital manometer from Accutherm, Melbourne, Australia was used to measure transmembrane pressure (TMP) changes. The membrane was placed in a custom made filtration module made from stainless steel, giving an effective membrane area of 2.5 cm^2^. A programmable Vulcan 3-550PD NEY furnace, (Extech Equipment, Victoria, Australia) was used for heat treating the membrane after coating with TiO_2_. A peristaltic pump (Masterflex 7592-45, Cole-Parmer, Vernon Hills, IL, USA) was used to drive the feed through the membrane in dead-end mode. The amount of permeate collected was measured by an electronic balance (FX-3000i WP, A&D Company Ltd., Seoul, South Korea) with real time monitoring software. A sonic bath (Soniclean 500HT, Transtek Systems, Melbourne, Australia) was used to ultrasonically clean the membranes before coating, as well as to remove air bubbles in the coating suspension.

### 2.3. Preparation of Membranes

The sintered glass discs were first cleaned by washing in acetone, ethanol then water in a sonic bath to remove loose particles and possible contaminants. Each sonic wash was 20 min long. The last wash was followed by deionised water (DI water) rinsing and drying in a fan-forced oven at 80 °C for 3 h. The washed discs were weighed, labelled and stored in an air tight container until use.

To ensure coating on only one side of the membrane, one side was covered with masking tape. The coating suspension was prepared by adding 2 g of Evonik P25 TiO_2_ to 60 mL of 70/30% (*v*/*v*) water/methanol water acidified to pH 3 with nitric acid. The suspension was sonicated for 20 min followed by magnetic stirring for 2 h before the commencement of coating.

The disc was then dipped into the suspension using a custom made mechanical device and withdrawn at a dipping/withdrawal speed of 2 cm/min. The process was repeated three times. The coated membranes were then air dried over 12 h and the tape carefully removed, followed by heat treatment to 450 °C at a heating rate of 1 °C/min in a programmable muffle furnace. The temperature was held at 450 °C for 2 h, and then cooled to room temperature at 1 °C/min. The membranes were then washed with DI water and oven dried at 80 °C for 5 h. The membranes were weighed before and after coating to determine the amount of TiO_2_ that was immobilised on the surface.

### 2.4. Membrane Characterisation and Chemical Analysis

Fourier transform infrared spectroscopy (FTIR) analysis was used to explore surface functional groups and was performed with a Perkin Elmer Frontier FTIR Spectrometer equipped with an attenuated total reflectance (ATR) accessory. The crystal structure of the photocatalyst after coating was confirmed by powder X-ray diffraction (XRD) using a Rigaku Mini Flex 600 diffractometer operating with CuKα (*λ* = 1.54060 Å) radiation at 15 mA and 40 kV with a Ni filter. The analysis range was 20°–80° 2θ with 0.02° step and 1.2 s acquisition for steps. The step time was chosen to adequately obtain a good signal to noise ratio in the mean reflections of (1 0 1) and (1 1 0) planes, which are the two main anatase and rutile planes of TiO_2_ [10]. The substrate was ground by mortar and pestle before XRD analysis. The pore size of the substrate was determined by capillary flow porometry using a Quantachrome Porometer 3 GZ series. The method involves measuring nitrogen gas flow as a function of TMP through the dry and wetted membrane. The pore size is then calculated using the Washburn equation. The wetting liquid was Porofil from Quantachrome Corp., Boynton Beach, FL, USA. The absorbance of the sMO solution at 642 nm was measured by a UV-Visible-Biochrom Libra 522 UV-visible spectrophotometer. Total organic carbon (TOC) of BSA was determined by a Shimadzu TOC-V CSH analyzer.

### 2.5. Degradation of Screened Methyl Orange

The developed membranes were tested for photocatalytic activity under several configurations in UV light. The naked UV lamp had an intensity of 2.5 mW/cm^2^ at 365 nm and a distance of 10 cm as measured by the UV irradiance meter. The membranes were placed in a beaker with 100 mL 0.01 mM solutions of sMO and the discoloration of the dye monitored by UV-Visible light absorption measurements at 642 nm of 1 mL samples withdrawn by a micropipette at 30 min intervals. The membrane was placed such that only a thin layer of liquid was above the membrane. The first hour of each experiment was carried out in the dark to allow adsorption of the dye onto the membrane. The degradation experiments carried out are summarised in Table 1.

The pseudo first order rate constant of the dye degradation was calculated using the Langmuir-Hinshelwood equation:

(1) $$\ln\left(\frac{C}{C_{0}}\right)=-kt$$

where *C*_0_ is the initial concentration of dye, *C* is the concentration in mmol/L at time *t* (min), and *k* (min^−1^)is the rate constant [11].

### 2.6. Filtration of Humic Acid and BSA Rejection Tests

**Figure 2:**
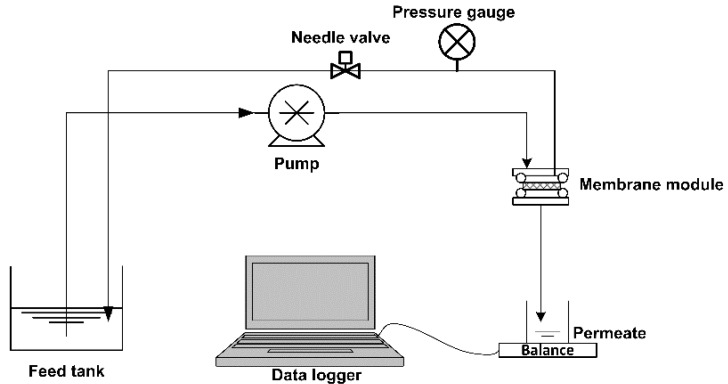
Schematic representation of the filtration setup.

A 20 mg/L HA solution prepared by the appropriate dilution of a previously prepared stock solution was used as the feed. Generally, the concentration range of humic substances in surface and ground water is 20 μg/L–30 mg/L [12]; therefore, 20 mg/L was specifically chosen to fall within the upper region of this range to represent a more challenging wastewater where there is a stronger need for membrane cleaning. To prepare the stock solution, 6 g of HA was mixed in 2 L of deionized water over 2 days by aid of a magnetic stirrer. Suspended solids were removed by vacuum filtration through a 0.45 µm membrane filter (ADVANTEC, Tokyo, Japan). The filtration setup is depicted in Figure 2, and it consisted of a feed tank, a peristaltic pump, membrane module, needle to close the retentate line such that the filtration mode was dead-end, pressure transducer to measure the change in transmembrane pressure (TMP), and a permeate collection tank placed on a balance connected to a data logger. The experiments were carried out at a constant flux of 450 L m^−2^ h^−1^. The membrane was first compacted with simulated tap water (100 mg/L NaCl solution) for 30 min, followed by filtration of the 20 mg/L HA solution for 30 min. Simulated tap water was used instead of real tap water so that the actual composition of the water was known, controllable and replicable. The membrane was then removed from the module and cleaned by either UV exposure under the lamp for 30 min, chemical cleaning using a 1% NaOH solution and 0.5% NaOCl, or simple rinsing in distilled water. After the cleaning process, the membranes were reloaded onto the module and the membrane recovery determined by measuring the new TMPs while continuing HA filtration for 30 min, cleaning and another HA filtration cycle. The cleaning methods used are summarised in Table 2.

The apparent fouling rate, *r*, in kPa/min between two adjacent cleaning events was calculated as:

(2) $$r=\frac{P_{max}-P_{min}}{\Delta t}$$

where *P_max_* is the TMP value immediately after the second cleaning event, *P_min_* is the TMP value immediately after the first cleaning event, and ∆*t* is the time between these two recorded TMP values.

The efficiency of each cleaning event was calculated as:

(3) $$E=\frac{P_{before}-P_{after}}{P_{before}-P_{0}}\;\times \;100\%$$

where *P_before_* and *P_after_* are the TMP values immediately before and after the cleaning event, and *P*_0_ is the pressure required to overcome the intrinsic membrane resistance [13].

To determine the selectivity of the substrate and prepared membrane, a 50 mg/L BSA solution was prepared by appropriate dilution of a 1 mg/mL BSA stock solution containing 1 mM CaCl_2_ and 7 mM NaCl in DI water. The BSA solution was then filtered through the uncoated substrate as well as the prepared membrane. The change in TOC was measured and the rejection, *R*, is calculated by

(4) $$R=1-\frac{C_{p}}{C_{f}}\;\times \;100\%$$

where *C_p_* and *C_f_* are the TOC concentrations in the permeate and feed, respectively.

## 3. Results and Discussion

### 3.1. Membrane Characterisation

**Figure 3:**
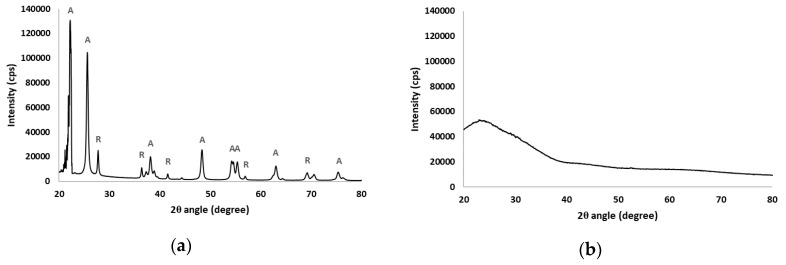
The X-ray diffraction (XRD) patterns of the (**a**) TiO_2_ photocatalyst; (**b**) the substrate with markers to indicate the Bragg peaks associated with the anatase (A) and rutile (R) phases.

The average pore size of the substrate as measured by porometry was 1.4 µm. After coating with TiO_2_, the normalized weight of the coating was measured on the membranes to be 4 ± 0.2 mg/cm^2^. The results of the XRD analysis of the photocatalyst and substrate are shown in Figure 3.

The diffraction pattern of the TiO_2_ immobilized on the membranes shows that the anatase phase is predominant, while the rutile phase is also present [14]. Typical anatase peaks include 25.7° (1 0 1), 38.1° (0 0 4), 48.5° (2 0 0), 54.3° (1 0 5), 55.3° (2 1 1) and 63.0° (1 1 8) [15,16], while rutile peaks appeared at 27.8° (1 1 0) [17], 36.4° (1 0 1), 41.6° (1 1 1), 57.1° (2 2 0) and 69.3° (3 0 1) [14]. The substrate is amorphous, therefore no distinct crystalline phases were detected.

**Figure 4:**
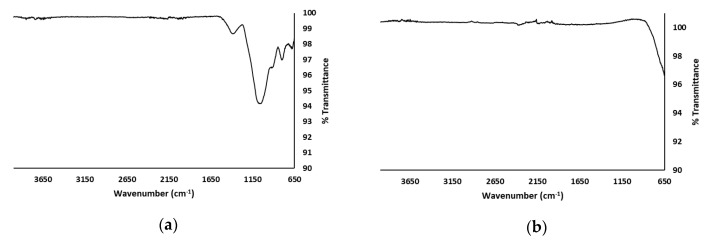
Fourier Transform Infrared (FT-IR) spectrum of the: (**a**) sintered glass substrate and; (**b**) P25 powder.

Figure 4 shows the Fourier Transform Infrared (FT-IR) spectrum of the sintered glass substrate and the P25 photocatalyst.

Borosilicate glass is a composite of the three network forming units whose proportions depend on each particular type of glass. The units are trigonally coordinated boron (BO_3_), tetrahedrally coordinated boron (BO_4_), and tetrahedral SiO_4_ structural units. It can also consist of network modifiers such as alkali and or alkaline earth metal oxides. The peak appearing at 680 cm^−1^ is assigned to the bending vibrations of Si–O–B bridges. The peak at 920 cm^−1^ is due to the stretching vibrations of B–O bonds in tetrahedral BO_4_ units. The band between 1000–1120 cm^−1^ is thought to arise from overlapping contributions of silicate and borate groups containing BO_3_ and BO_4_ units. The absorption band which peaks at 1379 cm^−1^ is attributed to the B–O stretching vibrations of polymerized BO_3_ units. Broad bands which normally appear from 2200 cm^−1^ and extend beyond due to OH, water and hydroxyl groups, were not observed, mainly due to the heating process which drives out water. The barely noticeable peaks in this region are attributed to the stretching vibrations of O–H bonds which are formed at non-bridging oxygen sites [18,19]. FT-IR thus confirmed that the substrate consists of the borosilicate glass functional units. For the P25 samples, no significant peaks were observed.

### 3.2. UV Intensity

The intensity of the UV radiation at 365 nm passing through the membrane substrate was measured at 0.45 mW/cm^2^ at a distance of 10 cm from the light source. In contrast, 0.00 mW/cm^2^ was detected from a similar substrate with the same dimensions made from α-Al_2_O_3_. The sintered glass substrate is therefore better at conducting light than typical alumina substrates commonly used to make ceramic membranes.

### 3.3. Degradation of Screened Methyl Orange

**Figure 5:**
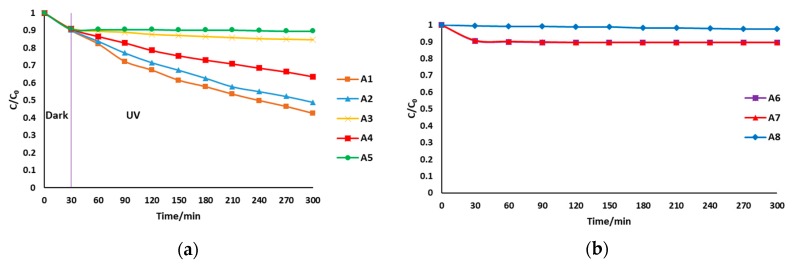
Dye concentration over time (**a**) in the presence of ultraviolet (UV); over the active layer (A1), through the substrate (A2), reflected (A3), through the substrate but reflection eliminated (A4), uncoated membrane (A5). (**b**) Adsorption by coated membrane (A6), adsorption by uncoated membrane (A7) and photolysis (A8).

Figure 5 shows relative changes of dye concentration with time for the various batch experiment setups. When light was directed onto the coated surface, about 58% of the dye had been degraded after 5 h (A1). This configuration can work well in cases of low turbidity waters, or low concentration dyes where there is minimal absorption of radiation by organic molecules or scattering and attenuation of radiation by minute particles present in the water. It is also of interest to note that the depth of the liquid above the coated layer was just 2 mm, therefore, no significant absorption of radiation by organic molecules would be expected. To address these limitations, UV directed through the substrate was investigated. After 5 h, the dye degradation percentage was 52% (A2). Although lower than when the UV was directed to the active layer, this configuration can be useful in turbid waters or high concentration organic solutions as mentioned before. The kinetics of screened methyl orange degradation thus showed that UV light could be successfully directed through the substrate to initiate photocatalytic reactions on the coated surface.

Table 3 shows the pseudo first order reaction rates of dye degradation through directing UV over the active layer (A1) and through the substrate (A2). The dye degradation in the other setups does not fit pseudo first order kinetics.

With regards to A2, there is the possibility that the apparent dye degradation was simply due to light reflected by the base of the reaction vessel. To remove any doubt, A3 was setup to determine the effect of reflected light on dye degradation, and about 11% degradation in 5 h was observed. This was almost equal to the apparent loss in dye concentration by adsorption to the membrane only (A6), showing that reflection is not a factor in the experiments. The apparent decrease in the degradation in A4 was due to the fact that the entire radiation from the lamp could not be directed onto the top of the membrane substrate where a collimated UV source would be more successful. Both the uncoated (A6) and coated membranes (A7) had the same dye adsorption capacity, which shows that the TiO_2_ layer’s main role was more to facilitate photocatalytic degradation than adsorption. An uncoated membrane in the presence of UV (A5) resulted in about 11% dye degradation, which shows that the photocatalytic layer plays an important role in the dye degradation. The amount of dye degraded by photolysis was less than 2.5% (A8). The largest contributor to dye degradation was therefore photocatalysis, followed by adsorption.

The rate constant obtained when directing light through the substrate is comparable to other results in the literature in Table 4. In all literature cases, UV was directed over the active layer. The configuration used in this study can therefore give dye degradation rates which are comparable to those in the conventional configuration, while also utilizing the advantages mentioned earlier.

### 3.4. Membrane Selectivity and Regeneration after HA Fouling

The BSA rejection of the substrate (measured by TOC) was found to be 11%, while that of the TiO_2_ coated membrane was 25%. Although BSA rejection increased slightly after coating the substrate, it is still low, indicating the membrane filtering in the microfiltration range.

**Figure 6:**
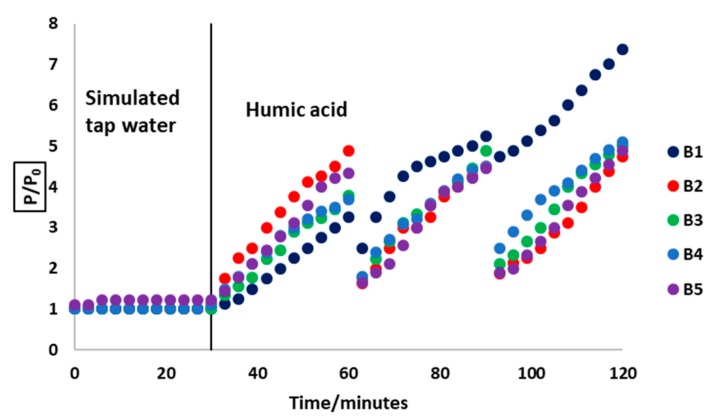
The variation of normalized pressure during filtration and between the cleaning procedures in which no cleaning was employed (B1); UV was applied over the active layer (B2); UV was directed through the substrate (B3); membrane was rinsed in DI water (B4); and membrane was rinsed in chemicals (B5).

Figure 6 shows the relative pressure change over the course of filtration of HA and simulated tap water. At the initial stage of simulated tap water filtration, there are no significant pressure changes due to the absence of foulants. The observed TMP is necessary to overcome the intrinsic membrane resistance. The value of P_0_ was 13 ± 2 kPa for the membranes. As soon as the feed is changed to a HA at 30 min, the TMP starts to rise due to fouling on the membrane surface. When there is no cleaning of the membrane that is carried out (B1), there is only a small pressure relief when the system is opened at 60 and 90 min. However, as soon as the filtration is restarted, the TMP quickly rises to values that are higher than before the system opening event. By the end of the 120 min filtration period, the TMP in B1 rises by more than 700% since the start of the filtration process. In contrast, cleaning the membrane by exposing the active layer to UV (B2) resulted in significant restoration of the membrane such that the final TMP is about 500% of the initial. This is also the case with UV exposure through the substrate (B3) and chemical cleaning (B5). Rinsing the membrane in DI water (B4) was the least effective in membrane regeneration. It is suggested that HA molecules penetrate the membrane pores, making it impossible to remove by simple water rinsing [23]; therefore, methods which breakdown the HA molecules would be more effective in mitigating fouling. Each UV cleaning step brings the TMP very close to the initial pressure, and the pressure rise thereafter does not reach the levels seen in the case where no cleaning takes place. UV exposure thus facilitates the photocatalytic degradation of HA molecules deposited on the membrane, which helps to regenerate the membrane.

Table 5 shows the apparent fouling rates that depict the different rates of foulant buildup on the membrane surface between the two cleaning events. Directing UV over the active layer (B2) resulted in the lowest rate of fouling, as was chemical cleaning (B5). This was closely followed by directing UV through the substrate (B3). Rinsing the membrane in DI water (B4) was the least effective in reducing the fouling rate since it had the closest rate to when no cleaning method was employed (B1).

As shown in Table 6, UV exposure on the active layer gives an 84% cleaning efficiency compared to 83% for chemical cleaning and 72% through the substrate for the first cleaning cycle. The second B2 cycle gives decreased cleaning efficiency because the non-reversible fouling layer shields radiation from fully accessing the photocatalytic sites. However, when UV is directed through the substrate in B3, there is only a slight decrease in efficiency, because the radiation path is still relatively free from interfering HA molecules. In long term use, the principle can be useful in maintaining the efficiency of the photocatalytic membrane regeneration process. Since the concept has been proved to work ex-situ, it is important to verify it in situ for longer term fouling mitigation. If a UV source, such as LED lights, is incorporated into the module, such that it can illuminate the photocatalyst through the substrate, continuous photocatalytic degradation of foulants can take place. The filtration process can therefore continue for longer, minimizing or even eliminating any form of physical or chemical cleaning.

The membrane was fabricated on top of a low cost, readily available sintered glass disc substrate for proof of principle testing. However, in considering the potential cost of the membranes for full scale water treatment, a substrate geometry and size suitable for practical applications in filtration is unknown and is the subject of future studies.

## 4. Conclusions

A light-conducting photocatalytic membrane was successfully prepared by dip coating TiO_2_ onto a sintered glass substrate. The membrane could conduct sufficient UV radiation to facilitate the photocatalytic degradation of sMO. It was shown to be capable of conducting UV for the purposes of mitigating HA fouling ex situ. Directing UV over the active layer showed the best membrane regeneration efficiency in the first cycle, followed by chemical cleaning. However, directing UV through the substrate had the best maintenance of efficiency across two cleaning cycles. The method is therefore of interest for further study during in-situ membrane cleaning over prolonged filtration periods.

## Figures and Tables

**Table 1:** Description of the screened methyl orange (sMO) degradation experiments.

Designation	Description	Purpose
A1	Coated membrane. Coated side facing UV source.	To determine effect of shining UV directly onto active layer.
A2	Coated membrane. Coated side facing away from UV source.	To determine the effect of transmitting light through the substrate.
A3	Coated membrane. Coated side facing away from UV source. Top part of membrane covered with aluminium foil.	To determine whether the apparent photocatalytic activated is due to light reflected from the base of the beaker, rather than light passing through the filter.
A4	Coated membrane. Coated side facing away from UV source. Every area of the beaker blocked except the top part of the membrane.	To focus the light source onto the filter, to determine whether it can transmit light that is sufficient enough to trigger photocatalytic reactions.
A5	Uncoated filter, in the presence of UV light.	To eliminate photocatalytic effect.
A6	Filter coated with P25, without UV light.	To determine adsorption property of coated membrane.
A7	Uncoated filter, without UV light.	To determine adsorption property of uncoated membrane.
A8	UV light only.	To determine the extent of photolysis.

**Table 2:** Description of the cleaning methods used to regenerate the membrane after fouling.

Designation	Description of Cleaning Method
B1	No cleaning employed
B2	UV exposure over the active layer
B3	UV exposure through the substrate
B4	Rinsing in DI water
B5	Rinsing in NaOH and NaOCl solutions

**Table 3:** Pseudo first order kinetics of the sMO degradation.

Configuration	UV Application Method	k (min^−1^)	R^2^
A1	Over active layer	0.0030	0.9869
A2	Through substrate	0.0025	0.9871

**Table 4:** Comparison of first order kinetics of directing UV to literature values.

Membrane	Target Compound	k (min^−1^)	R^2^	UV Intensity (mW/cm^2^)	Reference
TiO_2_/PMMA	∼0.01 mM MB	0.003	-	1.1	[20]
TiO_2_/PES	∼0.03 mM MO	0.004	0.99849	-	[17]
TiO_2_/GO-Psf	∼0.2 mM MB	0.004	-	-	[21]
TiO_2_/Fibreglass	∼0.02 mM MB	0.004	-	-	[11]
TiO_2_/SiO_2_	∼0.01 mM MB	0.006	0.99	5	[22]
TiO_2_/Sintered glass	0.01 mM sMO	0.003	0.9871	0.45	This work

**Table 5:** Apparent fouling rate.

Designation	Cleaning Method	Fouling Rate (kPa/min)
B1	No cleaning employed	0.60
B2	UV exposure over the active layer	0.07
B3	UV exposure through the substrate	0.10
B4	Rinsing in DI water	0.23
B5	Rinsing in chemical solutions	0.07

**Table 6:** Cleaning efficiencies of each method.

Designation	Cleaning Method	Cleaning Efficiency %	
		Cycle 1	Cycle 2
B1	No cleaning employed	33	12
B2	UV exposure over the active layer	84	75
B3	UV exposure through the substrate	72	71
B4	Rinsing in DI water	70	57
B5	Rinsing in chemical solutions	83	77
